# Endophytic Bacterial Community Structure and Function Response of BLB Rice Leaves After Foliar Application of Cu-Ag Nanoparticles

**DOI:** 10.3390/nano15110778

**Published:** 2025-05-22

**Authors:** Weimin Ning, Mingxuan Li, Lei Jiang, Mei Yang, Maoyan Liu, Yong Liu

**Affiliations:** 1 Agricultural Science College, Xichang University, Xichang 615000, China; limingxuan02085354@163.com (M.L.); leijiang411222@163.com (L.J.); yangmei202410@163.com (M.Y.); liu-mao-yan@foxmail.com (M.L.); 2 Longping Branch, College of Biology, Hunan University, Changsha 410125, China; 3 Key Laboratory of Pest Management of Horticultural Crop of Hunan Province, Hunan Academy of Agricultural Science, Changsha 410125, China

**Keywords:** endophytic bacteria, Cu-Ag nanoparticles, bacterial community, BLB, rice

## Abstract

Bacterial leaf blight (BLB) is a destructive disease caused by *Xanthomonas oryzae pv. oryzae* (*Xoo*). It has been proven that BLB adversely influences the growth and production of rice, resulting in substantial losses in yield. Nanoparticle–antimicrobial compounds possess excellent physicochemical properties, which have generated groundbreaking applications in protecting rice against BLB attacks. However, there is less research focused on the interaction between nanoparticles and the microbiome of BLB rice leaves, particularly the structure and function of endophytic bacteria, which are essential to plant health and pathogenesis. Therefore, the study explored how Cu-Ag nanoparticles influenced the endophytic bacteria’s composition and functions in healthy and BLB rice leaves. The data demonstrated that the relative abundance of beneficial bacteria, *Burkholderiales*, *Micrococcales*, and *Rhizobiales*, increased after the introduction of Cu-Ag nanoparticles on the leaves of BLB rice. The examination of PAL activity demonstrated that nanoparticles limited the spread of *Xoo* in rice leaves. Furthermore, endophytic community functional prediction demonstrated that nanoparticles may regulate the physiological process associated with potential stress resistance and growth-promoting function in the endophytic communities. This investigation may enhance the understanding of interactions between nanoparticles and the composition of rice endophytic microbiome, which can contribute to the exploration and application of nanomaterials in crop pathogen management.

## 1. Introduction

Rice (*Oryza sativa*) is a staple food and an important economic crop for the global population [1]. However, rice is easily threatened by various diseases during its growth and development, among which bacterial leaf blight (BLB) is particularly catastrophic [2]. BLB is initiated by the bacterium *Xanthomonas oryzae pv. oryzae* (*Xoo*) [3]. The disease can lead to considerable yield loss in the farmland, thus constituting an enormous threat to global food security and economic stability [4]. Currently, various strategies are employed to manage BLB in rice, including the development of resistant rice varieties, cultural behaviors, chemical measurements, and biological control. However, these strategies face considerable challenges [5]. The use of chemical control, particularly antibiotics, is limited due to the rise in bacterial resistance, environmental concerns, and potential adverse effects on human health [6,7,8]. Developing resistant varieties is time-consuming and costly, requiring continuous monitoring and the continuous creation of new resistant strains [9,10]. Biological control and cultural practices frequently show reduced effectiveness in some regions and require valuable workers’ investment [11,12]. Additionally, BLB can spread through multiple vectors, including wind, rain, soil, tools, and seeds [13]. Furthermore, its ability to adapt to environmental conditions further diminishes the effectiveness of these strategies.

Recent improvements in nanotechnology provide alternative approaches to control various plant pathogens [14,15]. The small size, larger surface area, high reactivity, and favorable volume-to-surface ratio of nanoparticles make them particularly effective in protecting rice from BLB [16,17]. A variety of nanomaterials have demonstrated that they can be effective against rice BLB, including ZnMgNPs, Ni-SiO2 NPs, Cu-NPs, Ag NPs, ZnO NPs, MnO2NPs, and MgONPs [18,19,20]. The use of nanoparticles can improve antibacterial effectiveness, decrease the reliance on traditional chemical pesticides, thus reducing the possibility of bacterial resistance and decreasing environmental pollution [21,22]. Furthermore, nanoparticles can enable targeted delivery and controlled release of antibacterial compounds, as well as improve plant immunity and growth [23,24,25]. For widespread and sustainable application of nanoparticles, it is essential to gain a comprehensive understanding of the interactions and mechanisms between nanomaterials and pathogens, plants, and environments, particularly the microbial communities of plants [26,27,28].

The leaves, stems, fruits, and roots of plants host various microbial communities, encompassing bacteria, archaea, and fungi [29]. These bacteria exist as endophytes and epiphytes in the plants. Most endophytes are beneficial microorganisms that can form a mutualistic relationship with plant tissues without causing harm to the plant host. They play a crucial role in enhancing plant health by suppressing pathogens, activating plant defense responses, promoting growth, and initiating systemic acquired resistance [30,31,32,33]. Zhang et al. [34] found that zinc oxide nanoparticles substantially improved soil nutrient content and influenced the root endophytic microbial diversity, specifically increasing the amount of *Nakamurella*, *Aureimonas*, *Luteimonas*, and *Sphingomonas*. These bacteria play a crucial role in helping soybeans mitigate aluminium toxicity. Sun et al. [35] synthesized the silver nanoparticles using endophytes. AgNPs with a maximum size of 31.34 nm demonstrated noteworthy antibacterial and antifungal properties, and AgNPs can effectively extend the storage life of cherry tomatoes. Ijaz et al. [36] synthesized melatonin-biogenic silica nanoparticles utilizing leaf extract, and a greenhouse study showed that the nanoparticles can mitigate tomato bacterial wilt disease and enhance beneficial bacterial communities in the rhizosphere.

In our earlier study, we demonstrated the antibacterial efficacy of peptides and bimetallic Cu-Ag nanoparticles supported on multiwalled carbon nanotubes (MWCNTs&CuNCs@AgNPs@P) against the rice BLB pathogen in vitro and in vivo [37]. This study utilized bacterial 16S rRNA gene amplicon technologies to analyze the community structure and abundance of bacteria in healthy and *Xoo*-infected rice leaves under Cu-Ag nanoparticle exposure. Meanwhile, the nutrient content and pathogen-related enzyme activities in rice leaf samples were also examined to investigate the fundamental mechanism of rice response to BLB disease. Our study can help to comprehend the altered strategies of endophytic microbe communities in diseased rice leaves in response to nanoparticle treatment.

## 2. Materials and Methods

### 2.1. Materials

Thiodiazole-copper was purchased from Longwan Chemicals Co., Ltd., Zhejiang, China; Nitric acid was purchased from Aladdin Biochemical Technology Co., Ltd., Shanghai, China; The rice cultivar and *Xanthomonas oryzae pv. oryzae.* (*Xoo*) were stored in the Key Laboratory of Pest Management of Horticultural Crop of Hunan Province, Hunan Academy of Agricultural Science; the MagPure Soil DNA LQ Kit was purchased from Magen Biotechnology Co., Ltd., Guangzhou, China; the Tks Gflex DNA Polymerase was purchased from Takara Biomedical Technology Co., Ltd., Beijing, China; the DNeasy PowerSoil kit was purchased from Qiagen N.V Co., Ltd., Hilden, Germany; the Qubit dsDNA Assay Kit was purchased from Life Technologies Pvt Ltd., Carlsbad, CA, USA.

### 2.2. The Synthesis of Nanoparticles

The synthesis of MWCNTs&CuNCs@AgNPs@P was based on our previous research [37]. Initially, the reaction mixtures of MWCNTs-COOH, polyethylene glycol, 1-(3-Dimethylaminopropyl)-3-ethylcarbodiimide hydrochloride (EDC), and N-hydroxysuccinimide (NHS) were subjected to centrifugation at 12,000 rpm for 20 min following 24 h of stirring. The precipitate (MWCNTs-COOH-PEG) was gathered and re-dispersed in sterile water. Subsequently, bovine serum albumin and CuSO_4_ were added to fresh sterile water, and then a slow addition of NaOH was made to obtain a pH of 12. MWCNTs-COOH-PEG and AgNO_3_ were then introduced to the mixture and stirred vigorously for 30 min. The hydrazine hydrate was added and stirred at 80 °C for 15 min and then stirred at room temperature for 20 min. Afterwards, the peptide solution, EDC, and NHS were incorporated, and the mixture was stirred again for 4 h. The reaction mixture was then filtered via dialysis membranes, and the resulting mixed solutions (MWCNTs&CuNCs@AgNPs@P) were utilized for subsequent analysis.

### 2.3. The Characterization of Nanoparticles

Fourier transform infrared spectroscopy (FTIR) of Cu-Ag nanoparticles was performed on a Nicolet 460 FTIR spectrometer (Thermo Fisher Scientific, Milwaukee, WI, USA) and a Spark 10 M multimode microplate reader. The morphology of MWCNTs&CuNCs@AgNPs@P nanoparticles was examined using a transmission electron microscope (TEM). The nanoparticles were disseminated in an ethanol solution, and the dilute dispersion underwent ultrasonication for 30 min. Subsequently, the nanoparticle solution was drop-cast onto a carbon-coated copper grid without discoloration. Then the grids were dried at room temperature and imaged via TEM. Samples were examined employing a TEM (JEM-F200, JOEL, Tokyo, Japan) under an accelerating voltage of 200 kV. For the scanning electron microscopy (SEM) characterization, MWCNTs&CuNCs@AgNPs@P nanoparticles were subjected to ultrasonication for 30 min. Following this, the nanoparticle solution was put on a conductive adhesive. The sample was treated with gold sputtering for a duration of 45 s by a sputtering coater (Quorum Technologies, Sacramento, CA, USA), with a gold spraying current set at 10 mA. Samples were viewed and photographed with a SEM (Sigma 300 VP SEM, Zeiss Gemini, Oberkochen, Germany) functioning at an accelerating voltage of 3 kV.

### 2.4. Plant Growth

After five minutes of sterilizing in ethanol solution, rice seeds were extensively washed using sterile water. Germination started on a wet plate for five days, with sterile water replaced once a day. Afterward, seedlings with uniform root and shoot sizes were chosen and transferred to soil. Rice was then transferred into a growth chamber (27 ± 2 °C day and night temperature and 60% humidity with 16 h light/8 h dark cycles). Rice was cultivated in a chamber for 40 days, and water was supplied every five days to maintain soil moisture levels.

### 2.5. Plant Spraying with Nanoparticles

The experiment was divided into six treatments with four replicates per treatment: healthy control (only sterile water was applied) (S12), healthy rice treated with 20 μL/mL MWCNTs&CuNCs@AgNPs@P (S13), healthy rice treated with thiodiazole-copper (diluted 500 times) (S14), BLB rice control (sterile water was applied) (S15), BLB rice treated with 20 μL/mL MWCNTs&CuNCs@AgNPs@P (TS16), and BLB rice treated with thiodiazole-copper (diluted 500 times) (S17). *Xoo* was initially cultured overnight on the NA plate in a bacteriological incubator at 25 °C and 200 rpm rotation. A single clone of *Xoo* was then chosen and placed into 50 mL of liquid medium and grown at 25 °C and 200 rpm. Finally, *Xoo* was resuspended to OD600 = 1, which was employed in the subsequent studies. The rice plants were inoculated with *Xoo* using the clipping method on fully developed leaves. The scissor tips were immersed in the *Xoo* suspension, and the leaf tip was cut approximately 2–3 cm away from the leaf. After 24 h, nanoparticles and thiodiazole-copper were applied to the rice seedlings using a hand-held sprayer until the rice was completely wet. Rice leaf samples for each treatment were collected 15 days post-spraying.

### 2.6. Phenylalanine Ammonia-Lyase (PAL) Activity Measurements

Direct spectrophotometric measurement was used to determine the PAL activity of rice leaf samples. This was defined as the rate of conversion from L-phenylalanine to trans-cinnamic acid at 290 nm [38]. A total of 0.1 g of fresh rice leaves was homogenized with 1 mL of extracted liquid. The extract was centrifuged at 10,000× *g* for 10 min at 4 °C. The supernatant was used as an enzyme source. After that, 0.2 mL of 40 mM phenylalanine and 0.4 mL of 100 mM Tris-HCl buffer are added to 0.2 mL of enzyme extract. The mixture was incubated at 37 °C for 30 min. The amount of trans-cinnamic acid synthesized was calculated using its extinction coefficient at 290 nm. Enzyme activity was expressed as the synthesis of trans-cinnamic acid. One activity unit was defined as a certain OD value change during a certain period.

### 2.7. Determination of Rice Leaves Mineral Element and Metal Contents

Phosphorus (P), silver (Ag), copper (Cu), and manganese (Mn) contents were determined in rice leaves. We randomly chose 3–4 cm lengths of the flag leaf from each treatment plant. Samples were placed in air-tight, sterile plastic bags. In total, 10 g of fresh rice leaf samples were oven-dried at 70 °C for 72 h under aseptic conditions. The collected samples were subjected to acid digestion for 12 h and diluted to a final volume of 10 mL after cooling for further analysis. Ag, Cu, and Mn content of the leaves’ digestion solution was determined by inductively coupled plasma mass spectrometry (ICP-MS; NexlON 1000G, Waltham, MA, USA) [39]. The contents of P were assessed via inductively coupled plasma-optical emission spectroscopy (ICP-OES; Agilent Technologies 5110, Santa Clara, CA, USA) [40].

### 2.8. Rice Leave Samples Harvest for Amplicon Sequencing

After 15 days of spraying, the rice leaves were harvested for 16S rRNA amplicon sequencing. We were wearing gloves and using scissors to collect rice leaf samples. For each treatment, we randomly chose rice plants, cut a 3–4 cm length of the flag leaf from each plant, and the rice leaf samples for each group were approximately 0.05 g. We immediately immersed them in a new tube containing 30 mL of sterile PBS buffer and placed them on ice. The PBS suspensions were shaken at 180 rpm for 15 min and then subjected to ultrasound for 10 min. Finally, the tube was centrifuged at 6000 rpm for 5 min to eliminate bacteria from the leaf tissue surface. Samples were cryopreserved in liquid nitrogen and maintained at −80 °C prior to use.

### 2.9. DNA Extraction and Amplicon Sequencing

Sterile rice leaf samples were cut into small pieces and powdered using a mortar and pestle in liquid nitrogen. Rice leaf samples were placed in a 1.5 mL Eppendorf tube containing sterile 0.1 mm diameter glass beads and TE buffer. The whole DNA of each leaf sample was extracted according to the manufacturer’s instructions. Agarose gel (1%) electrophoresis and a NanoDrop 2000 spectrophotometer were used to assess DNA concentration and integrity, respectively. Qualified DNA can be used directly for PCR amplification. To amplify the endophytic bacterial 16S rDNA regions in rice leaves, the pair of primers 343F (5′-TACGGRAGGCAGCAG-3′), 798R (5′-AGGGTATCTAATCCT-3′) was used [41]. The PCR reactions were carried out in 30 μL reactions using 15 μL of 2 × Gflex PCR Buffer, 1 μL 5 pmol/μL of forward and reverse primers, 0.6 μL 1.25U/μL Tks Gflex DNA Polymerase, and 50 ng of template DNA. The Qubit dsDNA assay kit was then used to quantify the PCR products after purification using Agencourt AMPure XP beads. Finally, the library was sequenced on an Illumina NovaSeq 6000.

### 2.10. Bioinformatics Analysis

The raw dataset was quality-trimmed using the cutadapt to exclude poor-quality readings, followed by denoising to fix sequencing errors properly. The “DADA2 plugin” was employed to collect the high-quality sequence reads [42]. QIIME2 was implemented to eliminate chimaeras, quality filter, and annotate raw sequences [43]. The software outputs the representative readings and the ASV abundance table. The beta diversity of microbial community structure among samples was visualized by principal coordinate analysis (PCoA) and nonmetric multidimensional scaling (NMDS) [44]. The biomarkers of rice were estimated through the random forest classification [45]. Furthermore, the abundance pattern diagrams are generated by analyzing the bacteria at the phylum, class, and order levels. At the functional level, differentially abundant Kyoto Encyclopedia of Genes and Genomes Orthology (KO) and Clusters of Orthologous Groups of proteins (COG) were performed [46,47].

### 2.11. Statistical Analysis

One-way analysis of variance (ANOVA) was utilized to assess the significance of the data. Tukey’s HSD test was utilized to identify significant differences across groups (*p* < 0.05). GraphPad Prism 10.3.0 and Origin 2024 were employed to create the graph.

## 3. Results

### 3.1. Morphology Characterization of Nanoparticles

Fourier transform infrared spectroscopy (FTIR) is a reliable analytical instrument used for the characterization of nanoparticles. The formulations of MWCNTs&CuNCs@AgNPs@P were studied by FTIR within the scanning range of 4000–500 cm^−1^. The nanoparticles exhibited functional groups characteristic of alcohols and phenols (–O–H) (3400–3600 cm^−1^), bending of aromatic C=C groups (1620–1680 cm^−1^), and a C–N stretching vibration (1540–1550 cm^−1^) [37]. The presence of these functional groups might promote the interaction between nanoparticles and bacteria. (Figure S1). TEM is an effective tool for analyzing the surface properties and morphological characteristics of nanomaterials. Two micrographs of Cu-Ag nanoparticles were acquired under different magnification circumstances. The majority of the particle forms have been found to be round and spherical. The two images illustrate nanoparticles with a narrow size distribution and display smooth surfaces (Figure S2). TEM mapping can provide a precise visualization of the distribution and proportion of each chemical element. The composition of MWCNTs&CuNCs@AgNPs@P includes atomic C, N, Cu, and Ag, and the nanoparticles demonstrated an average size of 79.24 nm [37]. The morphological details presented by the SEM corresponded with the information provided by TEM (Figure S3). Owing to their diminutive size and excellent dispersion, Cu-Ag nanoparticles demonstrated amazing effectiveness in protecting rice against *Xoo*. We used Cu-Ag nanoparticles to investigate their effects on the endophytic bacterial community of BLB rice.

### 3.2. Phenylalanine Ammonia-Lyase (PAL) Activity

**Figure 1:**
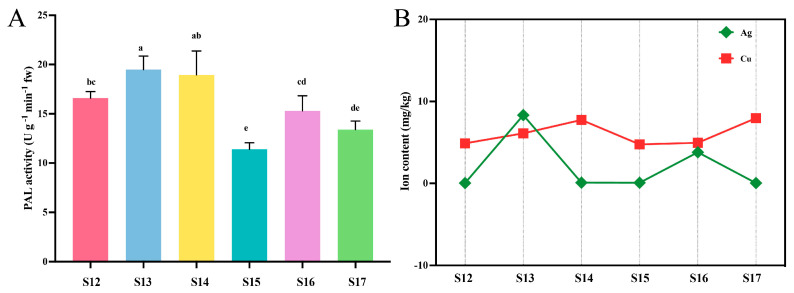
PAL activity (**A**) of leaves in *Xoo*-infected and healthy rice after being treated by bimetallic Cu-Ag nanoparticles and thiodiazole-copper in the greenhouse experiment. Ag and Cu (**B**) contents in Xoo-infected and healthy rice. Bars with different letters are significantly different. One-way ANOVA with Tukey’s HSD test (*p* < 0.05) and values are mean ± SD (standard deviation). Abbreviations: Healthy rice plants only treated with water (S12). Healthy rice treated with 20 μL/mL MWCNTs&CuNCs@AgNPs@P (S13), healthy rice treated with thiodiazole-copper (diluted 500 times) (S14), BLB rice control (sterile water was applied) (S15), BLB rice treated with 20 μL/mL MWCNTs&CuNCs@AgNPs@P (TS16), and BLB rice treated with thiodiazole-copper (diluted 500 times) (S17).

First, the impact of the application of Cu-Ag nanoparticles and thiodiazole-copper on PAL activity in rice leaves with and without *Xoo* infection was investigated. The results revealed that various treatments could change the PAL activity of rice. The PAL levels in the healthy rice treatments were significantly higher than in the infected rice group, while the control infected rice showed the lowest PAL activity. Notably, PAL activity increased in infected leaves following treatment with Cu-Ag nanoparticles (Figure 1A). These findings suggest that the changes in PAL activity may be attributed to the antibacterial properties of the synthesized nanoparticles.

### 3.3. Mineral and Metal Content in Rice Leaves

The implementation of nanomaterials can influence various compounds of plants, encompassing various nutrients and essential elements that are necessary for the growth and production of plants. The impact of various treatments on the leaf Ag and Cu content in healthy and diseased rice was investigated. The contents of Ag and Cu in leaves of healthy rice were different from those of rice attacked by *Xoo*. The Ag content was highest in healthy rice sprayed with Cu-Ag nanoparticles, while the Ag content in the leaves decreased with the invasion of *Xoo*. Similar trends were observed for Cu levels in rice across all treatments (Figure 1B). The essential micronutrient Mn, along with the required element P, was examined in rice leaves. The levels of P and Mn had increased in rice treated with nanoparticles, both in healthy rice and rice affected by *Xoo*, in comparison to the control healthy rice (Figure S4A,B). Our results show that foliar application of nanoparticles can regulate mineral nutrition.

### 3.4. Changes in Endophytic Microbial Members

There are eighteen rice leaf samples in this study, with the total raw reads ranging from 78,001 to 81,798. Following quality control, the number of high-quality reads ranged from 72,038 to 76,084. The number of ASVs present in each rice sample varies between 4 and 17. To obtain a better understanding of the dynamics of endophytic bacteria in healthy and diseased rice subjected to various treatments. The flower plot was employed to analyze the number of ASVs displayed as proprietary or shared in leaves. The analysis of all healthy rice leaves identified a total of four shared ASVs. A comparison of diseased rice leaf data revealed that four ASVs were shared (Figure S5A,B). Interestingly, the shared ASVs in the control healthy rice and three infected rice leaves, as well as in the control diseased leaf and the three healthy leaves, were all four. Further analyses were conducted in four different groups. More unique ASVs were identified in the control infected rice (eighteen) compared to the control healthy rice (four). With the application of Cu-Ag nanoparticles (eleven) and thiodiazole-copper (four) in diseased leaves, the unique ASVs decreased. Meanwhile, the unique ASVs were elevated following the treatment of healthy leaves with Cu-Ag nanoparticles (eleven) and thiodiazole-copper (seven) (Figure S5C,D). The analyses of ASVs reveal that the rice endophytic bacteria showed treatment-specific responses.

### 3.5. The Beta Diversity in Rice Endophytic Microbiota

**Figure 2:**
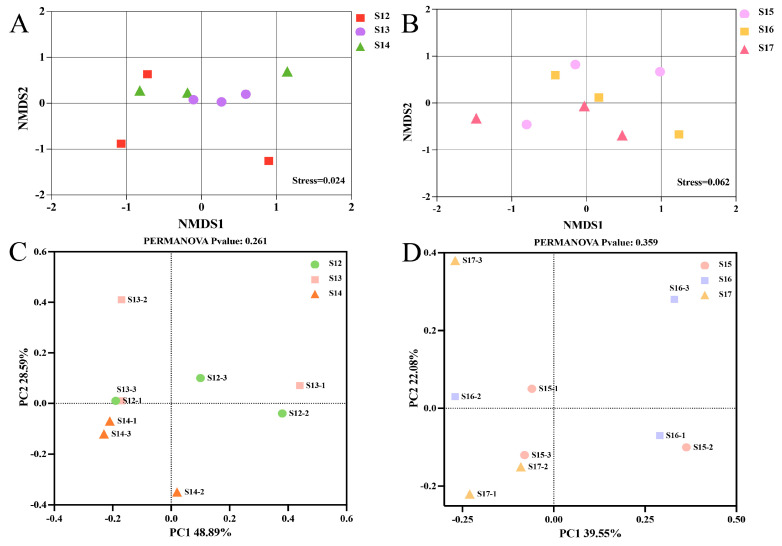
The beta-diversity of the rice leaves’ endophytic bacterial community. The two-dimensional NMDS plot of bacteria was visualized based on the Bray–Curtis matrix. The NMDS of group S12, S13, and S14 (**A**). The NMDS of groups S15, S16, and S17 (**B**). Principal coordinate analysis (PCoA) of bacterial community based on Bray–Curtis distance in the groups S12, S13, and S14 (**C**). The PCoA of groups S15, S16, and S17 (**D**).

To better understand the influence of Cu-Ag nanoparticles on the endophytic microbial population. The unconstrained principal coordinate analysis (PCoA), based on the Bray–Curtis distance, and a two-dimensional NMDS plot were utilised. There was a clear clustering between the control rice and treated samples. The leaves treated with nanoparticles exhibited a tendency to cluster together in both infected and healthy rice. Rice leaves treated with thiodiazole-copper exhibited a cluster tendency separate from the untreated samples (Figure 2A,B). The PCoA analysis revealed that the vertical axis accounted for 42.89% of the variation, whereas the horizontal axis explained 28.59% of the variation in the endophytic community of healthy rice leaves. The infected rice leaves exposed to the Cu-Ag nanoparticles and thiodiazole-copper exhibited variances of 39.55% for the first axis and 22.08% for the second axis (Figure 2C,D). Taken together, nanoparticles significantly affected the endophytic bacteria communities of the rice leaves.

### 3.6. Nanoparticles Charge the Leaf Endophytic Bacterial Microbiome

**Figure 3:**
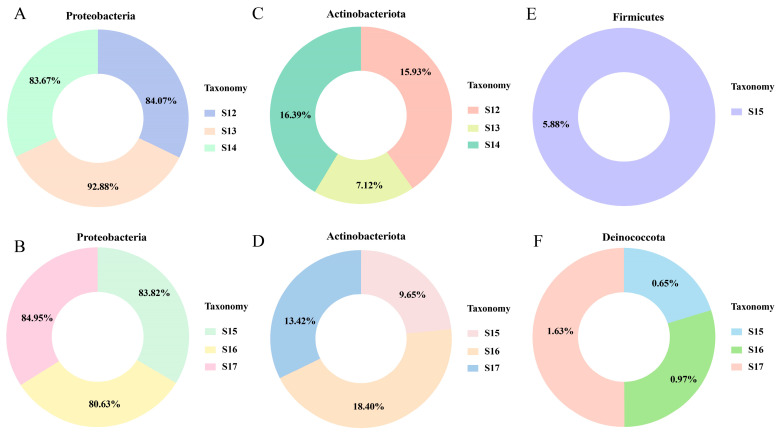
The structure of the endophytic bacterial communities in rice at the phylum level. The relative abundance of *Proteobacteria* in healthy rice (**A**) and *Xoo*-infected rice (**B**). The relative abundance of *Actinobacteriota* in healthy rice (**C**) and *Xoo*-infected rice (**D**). The relative abundance of *Firmicutes* in control *Xoo*-infected rice (**E**). The relative abundance of *Deinococcota* in *Xoo*-infected rice (**F**).

The 16S rRNA amplicon metabarcoding was performed to assess the modification of the leaf endophytic microbiome community in compliance with the application of Cu-Ag nanoparticles and thiodiazole-copper. The bacteria at the species, genus, family, order, class, and phylum levels in the phyllosphere microbial communities experienced alterations (Figure S6). At the phylum level, the bacterial communities of healthy rice leaves were predominantly characterized by two abundant phyla: *Proteobacteria* and *Actinobacteriota*. The relative abundance of *Proteobacteria* in healthy rice plants showed an increase after the NPs treatment, which substantially lowered the relative abundance of *Actinobacteriota*. Interestingly, the alterations observed in the thiodiazole-copper treatment were opposite to those of the two bacteria in the nanoparticle-treated groups (Figure 3A,C). Furthermore, in the infected rice leaves, *Proteobacteria* was the predominant phylum, and the next most dominant phyla were *Actinobacteriota*, *Firmicutes*, and *Deinococcota*. The incubation of nanoparticles led to a decrease in the relative abundance of Proteobacteria, whereas the relative abundance of *Actinobacteriota* and *Deinococcota* increased. The number of *Actinobacteriota* nearly doubled in the nanomaterial treatment compared to the control infected rice. Moreover, the diseased rice leaves treated with thiodiazole-copper showed an increase in the relative abundance of *Proteobacteria*, *Actinobacteriota*, and *Deinococcota* (Figure 3B,D). Additionally, *Firmicutes* were solely observed in control infected rice leaves (Figure 3E).

**Figure 4:**
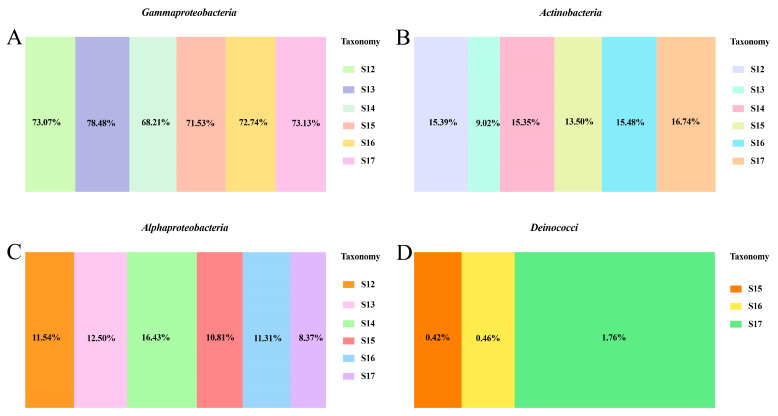
Relative species abundance at the class level of all rice leaf samples. The bacterial community abundance of *Gammaproteobacteria* (**A**), *Actinobacteria* (**B**), *Alphaproteobacteria* (**C**), and *Deinococci* (**D**).

An analysis of the endophytic bacterial community was carried out at the class level. It was observed that the predominant bacterial classes in all rice leaves were *Gammaproteobacteria*, *Actinobacteria*, and *Alphaproteobacteria*. The abundance of the three bacterial classes exhibited significant variations between treatments. The relative abundance of *Gammaproteobacteria* was reduced in the rice infected by *Xoo* compared to healthy control rice. In contrast, the relative abundance of *Gammaproteobacteria* showed an increase when the leaves were treated with Cu-Ag nanoparticles and thiodiazole-copper. The alteration in *Actinobacteria* corresponded with that in *Gammaproteobacteria*. The relative abundance of *Actinobacteria* was increased in diseased rice with the introduction of Cu-Ag nanoparticles and thiodiazole-copper. The invasion of *Xoo* diminished the number of *Alphaproteobacteria* compared to healthy rice, and the addition of nanoparticles effectively restores the level of *Alphaproteobacteria* near that in control healthy rice (Figure 4A–C). Additionally, *Deinococci* were found only in diseased rice leaves, with the highest numbers observed in the rice treated by thiodiazole-copper (1.76%) and the lowest in control diseased rice (0.42%). The proportion of *Deinococci* was 0.46% in the nanoparticle-treated group (Figure 4D and Figure S7A).

The effect of nanoparticles on the bacterial community in healthy and damaged rice leaves at the order level was also investigated. The top five bacterial orders found in all rice leaves were *Burkholderiales*, *Micrococcales*, *Rhizobiales*, and *Oceanospirillales*. The application of nanoparticles and thiodiazole-copper increased the relative abundance of *Burkholderiales*, *Micrococcales*, and *Rhizobiales* in diseased leaves. Notably, the amount of *Burkholderiales*, *Micrococcales*, and *Rhizobiales* in the nanoparticle group was more equivalent to that in the control healthy rice. The relative abundance of *Oceanospirillales* was increased following the invasion of *Xoo*, but *Oceanospirillales* showed a decrease when infected rice was treated with nanoparticles (Figure S7B). Furthermore, the influence of Cu-Ag nanoparticles on the bacterial community at the genus level was also investigated. The main bacteria discovered were *Comamonas* in all six rice groups; the introduction of *Xoo* decreased the abundance of *Comamonas*. The application of nanoparticles in diseased rice caused a significant increase in the relative abundance of *Comamonas*, surpassing the levels observed in healthy rice. The relative abundance of *Nesterenkonia* and *Afipia* decreased with the invasion of *Xoo*, while the use of Cu-Ag nanoparticles and thiodiazole-copper caused an increase in the levels of the two bacterial genera in diseased rice. Meanwhile, the utilization of nanomaterials decreased the relative abundance of *Pelomonas* and *Halomonas* in rice affected by *Xoo* (Figure S7C). The presented data indicated that NPs displayed different influences on the abundance of endophytic bacteria, which caused variations in the composition of the bacterial community between healthy rice and rice infected with *Xoo*.

### 3.7. Unique Bacterial Species in the Rice Leaves

**Figure 5:**
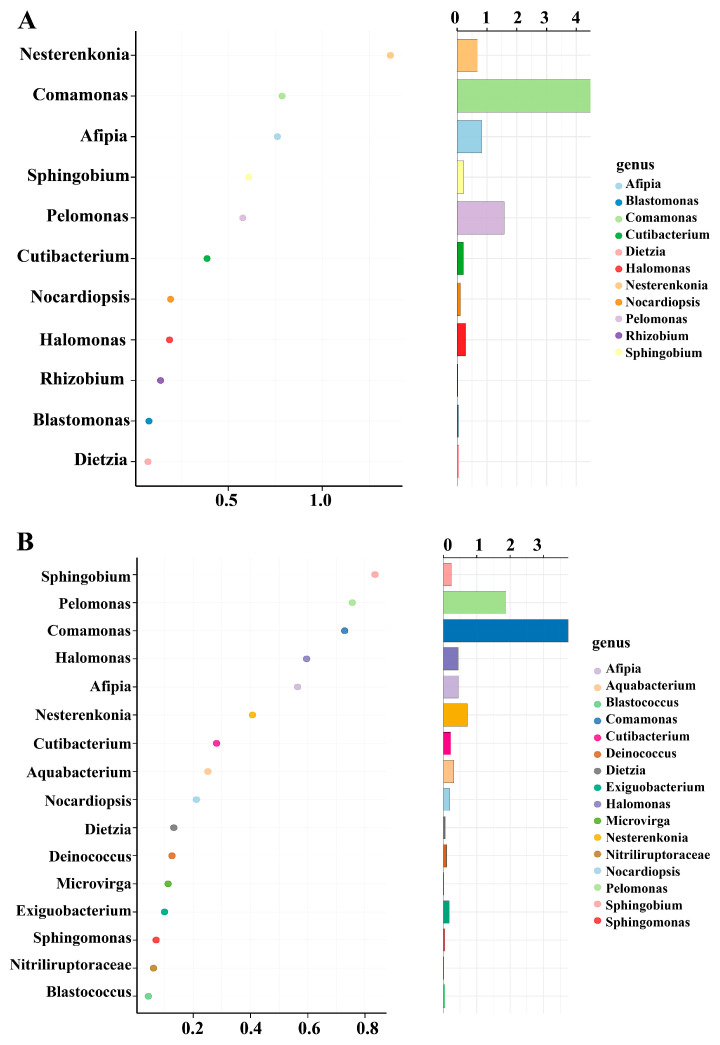
The Random Forest machine-learning model is utilized to identify endophytic bacteria biomarkers present in rice leaves. The different endophytic bacterial communities in healthy rice leaves (**A**) and diseased leaves (**B**). Bacterial taxa are ranked in declining order of significance at the genus level.

The random forest method can effectively identify the microbial communities that show substantial variations across different treatments. This approach was utilized to examine the feature biomarkers in rice leaves at the genus level. In the healthy rice group, 12 bacterial species showed significantly higher relative abundance in the leaves, while 16 bacterial biomarker taxa were notably abundant in leaves infected by *Xoo*. The bacterial genera *Nesterenkonia*, *Afipia*, *Comamonas*, and *Pelomonas* were predominant keystone taxa in healthy rice (Figure 5A). In contrast, four feature bacteria exhibited higher relative abundances in infected leaves, the biomarker taxa categorized as *Sphingobium*, *Pelomonas*, *Comamonas*, and *Afipia* (Figure 5B). The results indicate there are variations in keystone bacterial taxa between healthy and infected rice leaves. Furthermore, there were a higher number of bacterial genera present in diseased rice, which may be attributed to the application of Cu-Ag nanoparticles and thiodiazole-copper, potentially encouraging more microorganisms to combat the invasion of pathogens.

### 3.8. Changes in Endophytic Microbial Function

**Figure 6:**
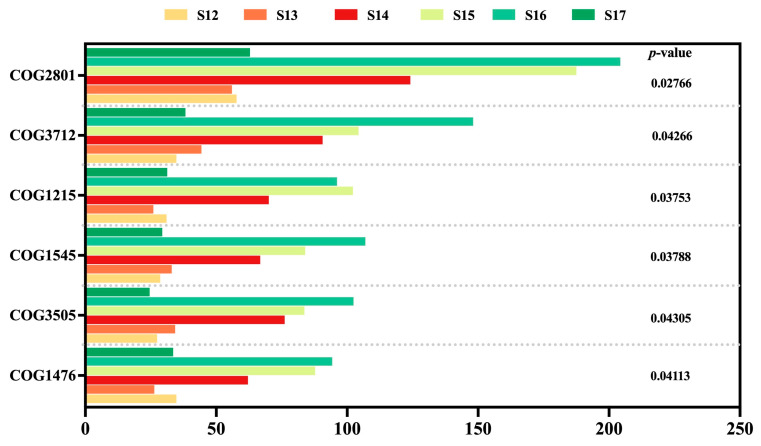
Differential abundance analysis of the cluster of orthologous groups of proteins COG functional genes of the endophytic microbiomes of healthy and infected rice leaves across Cu-Ag nanoparticles and thiodiazole-copper.

To further understand the functional prediction of endophytic bacterial communities in rice leaves, we utilized the PICRUST system to analyze the cluster of orthologous genes (COG) functional characteristics of microbiomes. The COG functional annotations demonstrated that the functions of DNA-binding transcriptional regulator (COG1476), type IV secretory pathway (COG3505), OB-fold protein (COG1545), glycosyltransferase (COG1215), protein ferR (COG3712), and IS3 family (COG2801) were distinctive from each rice leaf sample. The relative abundance of genes associated with glycosyltransferase diminished in both healthy and diseased rice treated with Cu-Ag nanoparticles. The other five functional genes were positively regulated by nanoparticles in the infected leaves (Figure 6). The variance of the abundance analysis indicates that the functional characteristics of rice leaves treated with nanoparticles differ between healthy and infected phases. The KO ortholog homologous function predictions for healthy rice leaf samples show that the introduction of Cu-Ag nanoparticles reduced the abundance of tam (K00598), DPM1 (K00721), catE (K07104), ydjE (K08369), ecfA2 (K16787), coaA (K00867), catE (K07104) and aapJ (K09969). Conversely, these functions were elevated with the addition of thiodiazole-copper in healthy leaves. Meanwhile, with the invasion of pathogens *Xoo*, the function of the microbial community in the endophyte of rice leaf samples had adaptive modifications. Nanoparticles enhanced the levels of fes (K07214), which were diminished by thiodiazole-copper. In the presence of nanoparticles, the levels of ABC.MN.S (K09818) and prdX (K19055) were suppressed (Figure S8A,B).

**Figure 7:**
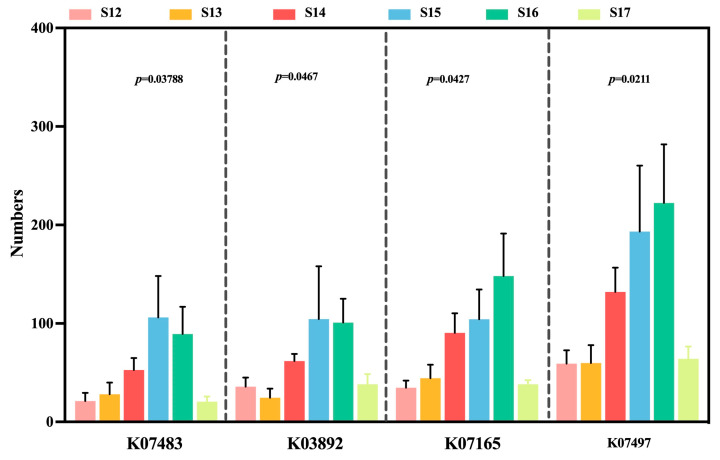
PICRUSt2 predicts the relative abundance of functional genes that exhibited significant differences among all rice leaves, utilizing the Kyoto Encyclopedia of Genes and Genomes Orthology (KO).

To enhance understanding of the functional changes in rice leaves following nanoparticle treatment, which may exhibit either a negative or protective influence on *Xoo* invasion. The findings indicated that four functions displayed significant variations in all rice leaf samples. The decrease was found in rice leaf samples regarding putative transposase (K07497), transposase (K07483), arsR (K03892), and fecR (K07165) following the invasion of *Xoo*. The levels of transposase increased in infected rice treated with nanoparticles, leading to inhibition of ArsR and fecR expression. The expression trends of these four genes were diminished in infected rice treated with thiodiazole-copper compared to the control diseased rice. However, thiodiazole-copper elevated the expression level of functions in healthy rice. Additionally, the varying expression levels of these functional genes were observed in healthy rice treated with Cu-Ag nanoparticles. The implementation of nanoparticles resulted in a higher relative abundance of transposase and fecR, whereas the level of ArsR showed a reduction in healthy rice (Figure 7). Our results showed that Cu-Ag nanoparticles increased the activity of functions associated with stress resistance in the rice leaves’ endophytic bacterial communities.

## 4. Discussion

In our previous experiments, we demonstrated that Cu-Ag nanoparticles have the ability to effectively protect rice from *Xoo*. However, the underlying role and mechanisms remain unexplored. To understand the mechanism of reducing rice BLB disease through the application of nanomaterials, we primarily investigate the impact of nanomaterials on enzyme activity and the endophytic bacteria community of rice. Plants have the ability to activate various defense enzymes and inhibitors that play a crucial role in combating pathogen attacks [48]. PAL is a catalytic enzyme that stimulates the transformation of Phe to cinnamic acid, which is the first step of the phenylpropanoid pathway [49]. Meanwhile, PAL is essential for the generation of various defense-related compounds such as phenols and lignins, and it also can help the elimination of ammonia [50,51]. In our study, the invasion of *Xoo* decreases the PAL activity in rice leaves, while the application of Cu-Ag nanoparticles and thiodiazole-copper substantially improved the PAL content compared with control infected rice. The results indicate that the application of nanoparticles strengthened the rice’s immune response. In our previous study, Cu-Ag nanoparticles had been demonstrated to diminish the disease severity index of BLB in rice leaves. Similar findings have been reported in other studies. Shams et al. [52] found that the application of ZnO nanoparticles substantially improves the PAL activity, which results in a decrease in Fusarium wilt caused by *Fusarium solani* in cherry tomatoes while simultaneously encouraging tomato growth. The increased level of PAL activity is a defensive response of plants against infections. This suggests that exposure to Cu-Ag nanoparticles could regulate PAL levels, thus possibly improving the ability of rice to respond effectively to *Xoo*.

Endophytic bacteria colonize either intracellularly or intercellularly in plant tissues. The communities of endophytic microorganisms living in plants show dynamic characteristics. The number, structure, and functions of endophytes depend on various plant features and environmental conditions, which include pathogenic strains, plant ages, conditions of climate, temperatures, plant genotypes, host exudates, nutrition, biotic and abiotic stress, together with the surrounding stations [32,53,54]. Understanding the interactions and biological functions of plant endophytic communities in response to pathogen pressure is crucial for maintaining a stable relationship between endophytes and plants.

This research investigates the microbiome existing in healthy and diseased rice leaves. The bacterial data for the leaves indicated that a lower number of unique ASVs were found in the control healthy rice compared to the control infected rice. This might be due to the invasion of the pathogen *Xoo* that influenced the microbial communities. The clustering patterns of the PCoA plot revealed the relationship and distinctions among various treatments. The findings showed that the endophytic microbial populations were separated following the utilization of Cu-Ag nanoparticles and thiodiazole-copper in both healthy and infected rice. Jiang et al. [55] found that the PCoA analysis of soil bacterial community displayed two distinct clusters in response to nanoparticle interactions with tomato bacterial wilt, demonstrating a variation of 21.6%. Furthermore, CuONPs can substantially decrease disease occurrence by altering the microbial community within the rhizosphere. Consequently, substantial differences have been observed in the bacterial community under various treatments.

The phyla associated with healthy and infected rice, when exposed to Cu-Ag nanoparticles and thiodiazole-copper conditions, were predominantly *Proteobacteria* and *Actinobacteriota*. It has been reported that *Actinobacteria* and *Proteobacteria* are commonly found in rice leaves. The relative abundance of the two bacteria is susceptible to change when rice varieties and environmental conditions are altered [56]. Notably, rice may exhibit different levels of abundance in response to pathogens [57]. Our study demonstrated that nanoparticles enhanced the number of *Actinobacteria* while decreasing the population of *Proteobacteria* in diseased rice. The phylum *Firmicutes* was only found in control infected rice leaves. A study found that *Firmicutes* were more abundant in the rhizospheres of plants growing in severe circumstances [58]. The findings indicated that *Proteobacteria* and *Actinobacteriota* were the predominant endophytic bacteria existing in both healthy and infected rice leaves treated with Cu-Ag nanoparticles and thiodiazole-copper.

The helpful interaction between plants and the microorganisms in the surroundings is essential for the growth and survival of the plants. The interaction among various beneficial microbes in plants can help them face various stress situations. Chavan et al. [59] found that silver nanoparticles and zinc oxide nanoparticles enhanced the relative abundance of *Burkholderiales* in soil, and a notable increase was discovered in soil treated with Ag nanoparticles. The order *Burkholderiales* are effective biodegraders of polychlorinated biphenyls, encompass natural biosynthetic gene clusters, and also show biosynthetic potential for the production of lipopeptides [60,61]. In our study, the application of Cu-Ag nanoparticles also led to higher numbers of *Burkholderiales* in infected rice leaves. Moreover, the relative abundance of *Micrococcales* and *Rhizobiales* also increased with the application of nanoparticles in *Xoo*-infected leaves. *Micrococcales* play an important role in metabolic functions, participating in carbon and nutrient metabolisms that enhance the metabolic activity of sediments [62]. Meanwhile, the fertilization treatments positively influence the abundance of *Micrococcales* [63]. *Rhizobiales* are specific microbial taxa that can convert atmospheric N_2_ into ammonia, contributing to N, C, and S cycles in nutrition, and can provide various nutrients, phytohormones, and precursors of essential metabolites [64,65]. Additionally, *Rhizobiales* show tolerance to organic pollutants, acidity, and various environmental stresses, and *Rhizobiales* can facilitate the survival of mangroves in intertidal regions [62]. These findings indicate that nanoparticles may interact with rice to modify the relative abundance of some endophytic bacterial groups on the leaves, potentially decreasing the incidence of rice BLB disease.

The arsenic acid-resistant (arsR) family consists of transcriptional regulatory factors that function as DNA-binding transcriptional repressors, and they are widely found among bacteria [66]. The arsR family plays a crucial role in various cellular processes, including the sustaining of metal ion homeostasis, the storing of excess heavy metal ions, the formation of biofilm, primary and secondary metabolism, responses to adverse stress, and virulence [67,68]. Zhi et al. [69] found that arsR is essential for regulating copper homeostasis in *Brucella*, and the elimination of arsR resulted in a reduction in virulence. In our study, with the implementation of nanoparticles in infected rice, there is a reduction in the BLB disease index and the levels of arsR, which is consistent with the positive correlation between lower arsR and diminished virulence activity. Ferric citrate-mediated iron transport is a key nonheme way for acquiring iron [70]. The fecR regulatory protein is essential for the cellular response to ferric citrate [71]. Notably, the levels of fecR are elevated with the introduction of nanoparticles in the *Xoo*-infected rice, which is significant because iron can enhance the production of the antibacterial compound tropodithietic acid [72]. The functional characteristics data indicate that nanoparticles significantly changed the microbial community functions of KO in the endophytic of rice.

In addition to predicting the homologous functions of KO orthologs, this study analyzed the COG ecological functions of rice microbial communities under various treatments. DNA-binding transcriptional regulators have the ability to sense stimuli in the environment and regulate the synthesis of specific genes [73]. These genes play an essential function in drug resistance, enzyme expression, metabolic pathways, bacterial pathogenicity, and the activity of antibacterial and antifungal [74,75]. The application of Cu-Ag nanoparticles led to an increase in DNA-binding transcriptional regulators in infected rice leaves. Additionally, Cu-Ag nanoparticles also upregulated the expression of type IV secretion systems, ferR, OB-fold protein, and IS3 family. These four functional genes are associated with horizontal DNA transfer, antibiotic resistance, DNA damage repair, and virulence in plant hosts [76,77,78]. The findings indicated that Cu-Ag nanoparticles enhanced the activity of functions related to stress resistance in the endophytic bacterial communities of rice leaves.

## 5. Conclusions

In summary, we investigated the influence of Cu-Ag nanoparticles on nutrient content, PAL enzyme activities, and the endophytic bacterial community structure and function in the healthy and *Xoo*-infected rice leaves. The findings indicated that nanoparticles exhibited various effects on the assembly of microbial communities in healthy and diseased rice. The application of nanoparticles in diseased rice resulted in an increase in the relative abundance of particular beneficial microorganisms and PAL activity. The findings strengthen the understanding of modifications in compositional structure, function, and multilateral interactions of the BLB rice endophytes under nanoparticle treatment, which benefits the exploration and utilization of nanoparticles in crop disease management and control.

## Data Availability

The raw high-throughput sequencing data were submitted to the NCBI (National Center for Biotechnology Information) database.

## Supplementary Materials

The following supporting information can be downloaded at: https://www.mdpi.com/article/10.3390/nano15110778/s1, Figure S1. Fourier transform infrared spectroscopy spectra of MWCNTs&CuNCs@AgNPs@P; Figure S2. Transmission electron microscope images of MWCNTs&CuNCs@AgNPs@P. The scale bars are 200 nm (A) and 50 nm (B); Figure S3. Scanning electron microscope (SEM) images of MWCNTs&CuNCs@AgNPs@P. The scale bars are 500 nm (A) and 110 nm (B); Figure S4. P (A) and Mn (B) contents of Xoo-infected and healthy rice leaves after being treated by bimetallic Cu-Ag nanoparticles and thiodiazole-copper in the greenhouse experiment; Figure S5. Venn diagram illustrating the shared and unique ASVs in groups S12, S13, S14 (A), groups S15, S16, S17 (B), groups S12, S13, S14, S15 (C), and groups S12, S15, S16, S17 (D); Figure S6. Taxonomic compositions at the annotation level of healthy and Xoo-infected rice leaves; Figure S7. The relative abundance of Bacilli in control *Xoo*-infected rice (A). The relative abundances of bacterial taxa at the order level (B) and the genus level (C) of the healthy and *Xoo*-infected rice leaves treated by Cu-Ag nanoparticles and thiodiazole-copper; Figure S8. The heatmap demonstrates the functional prediction of genes based on the Kyoto Encyclopedia of Genes and Genomes Orthology (KO) in healthy rice (A) and infected rice (B). Each row of the heatmap represents a specific gene, while each column represents an independent rice leaf sample.
