# Supplementary material for: Endophytic Bacterial Community Structure and Function Response of BLB Rice Leaves After Foliar Application of Cu-Ag Nanoparticles
Source: Nanomaterials (Basel). 2025 May 22;15(11):778. doi: 10.3390/nano15110778 (PMC12157765; doi:10.3390/nano15110778)
Supplement: Supplementary file 1 [file nanomaterials-15-00778-s001.zip › nanomaterials-3589067-supplementary.pdf]

nanomaterials

Supplementary Materials

Endophytic Bacterial Community Structure and Function Response of BLB Rice Leaves After Foliar Application of Cu-Ag Nanoparticles

Weimin Ning [1,2,*], Mingxuan Li [1], Lei Jiang [1], Mei Yang [1], Maoyan Liu [1] and Yong Liu [2,3,*]

[1] Agricultural Science College, Xichang University, Xichang 615000, China; limingxuan02085354@163.com (M.L.); leijiang411222@163.com (L.J.); yangmei202410@163.com (M.Y.); liu-mao-yan@foxmail.com (M.L.)
[2] Longping Branch, Biology College of Hunan University, Changsha 410125, China
[3] Key Laboratory of Pest Management of Horticultural Crop of Hunan Province, Hunan Academy of Agricultural Science, Changsha 410125, China
* Correspondence: ningweimin@hnu.edu.cn (W.N.); liuyong@hunaas.cn (Y.L.)

Academic Editor: Silvana Alfei

Received: 31 March 2025
Revised: 19 May 2025
Accepted: 20 May 2025
Published: 22 May 2025

Citation: Ning, W.; Li, M.; Jiang, L.; Yang, M.; Liu, M.; Liu, Y. Endophytic Bacterial Community Structure and Function Response of BLB Rice Leaves to Foliar Application of Cu-Ag Nanoparticles. *Nanomaterials* **2025**, *15*, 778. https://doi.org/10.3390/nano15110778

Copyright: © 2025 by the authors. Licensee MDPI, Basel, Switzerland. This article is an open access article distributed under the terms and conditions of the Creative Commons Attribution (CC BY) license (https://creativecommons.org/licenses/by/4.0/).

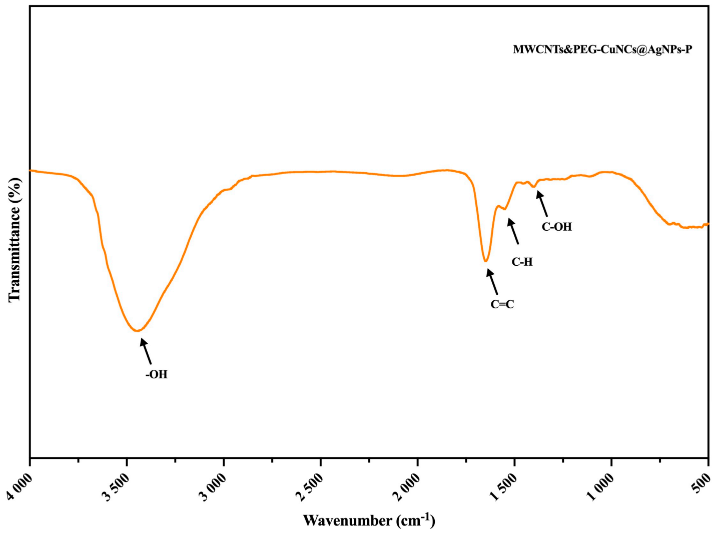

Figure S1. Fourier transform infrared spectroscopy spectra of MWCNTs&CuNCs@AgNPs@P [1].

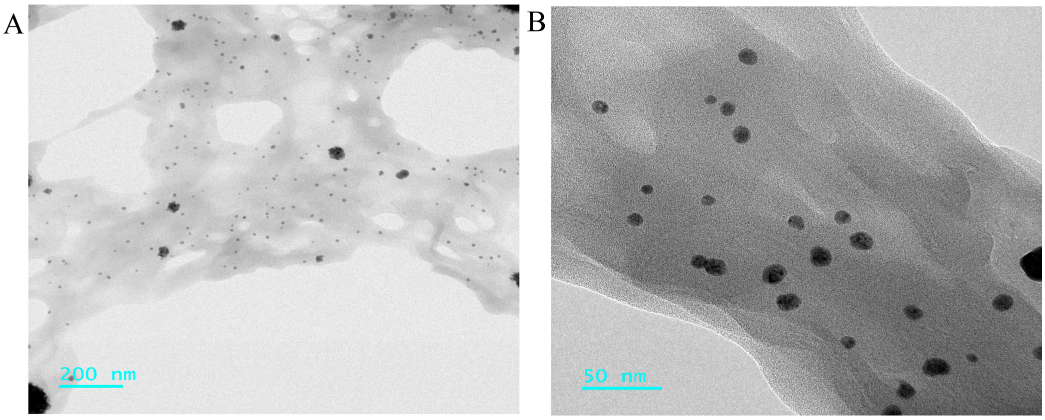

Figure S2. Transmission electron microscope images of MWCNTs&CuNCs@AgNPs@P. The scale bars are 200 nm (A) and 50 nm (B).

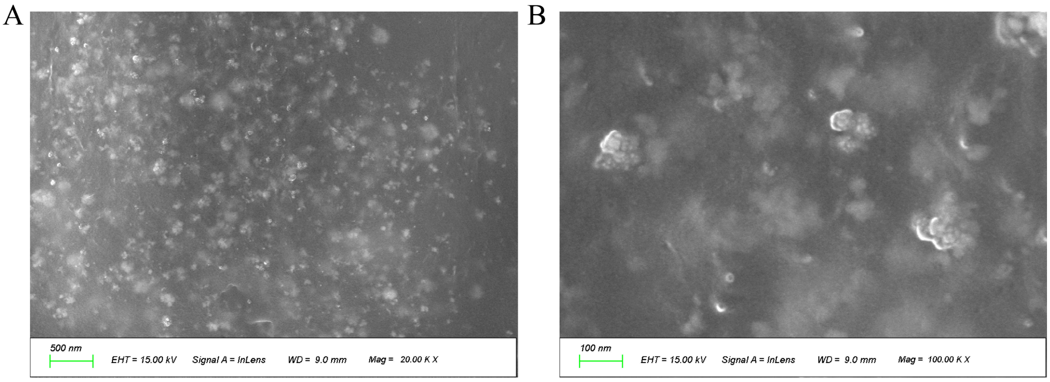

Figure S3. Scanning electron microscope (SEM) images of MWCNTs&CuNCs@AgNPs@P. The scale bars are 500 nm (A) and 110 nm (B).

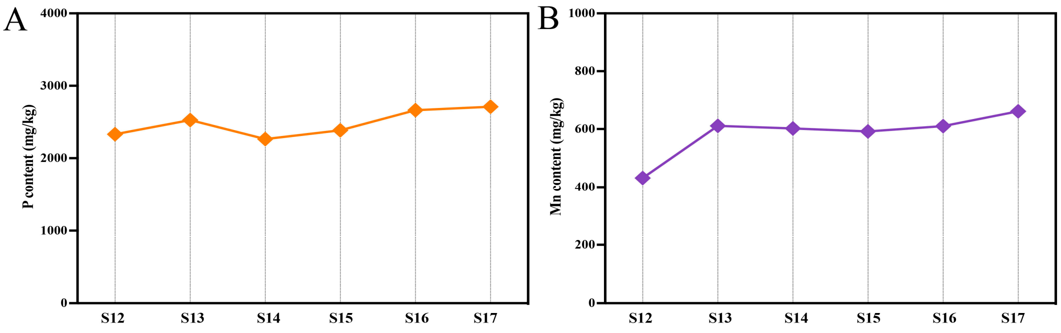

Figure S4. P (A) and Mn (B) contents of *Xoo*-infected and healthy rice leaves after being treated by bimetallic Cu-Ag nanoparticles and thiodiazole-copper in the greenhouse experiment.

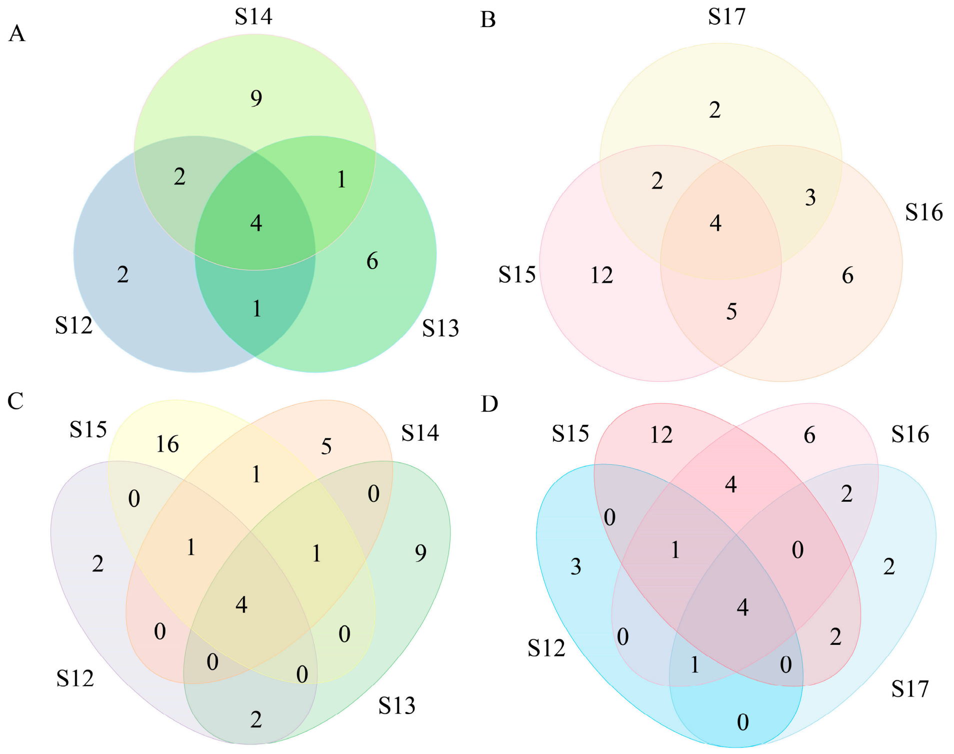

Figure S5. Venn diagram illustrating the shared and unique ASVs in groups S12, S13, S14 (A), groups S15, S16, S17 (B), groups S12, S13, S14, S15 (C), and groups S12, S15, S16, S17 (D).

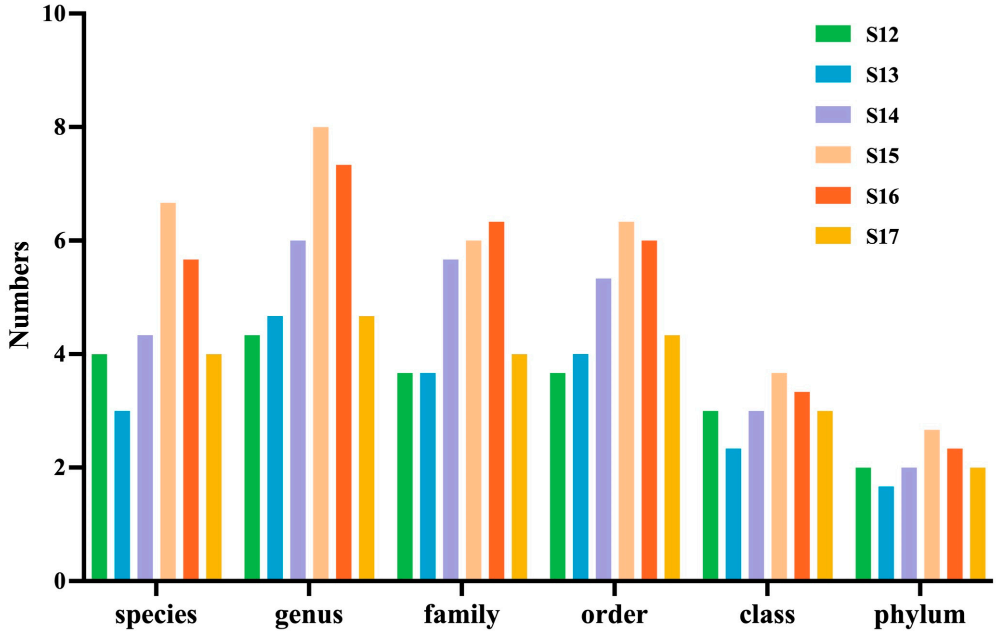

Figure S6. Taxonomic compositions at the annotation level of healthy and *Xoo*-infected rice leaves.

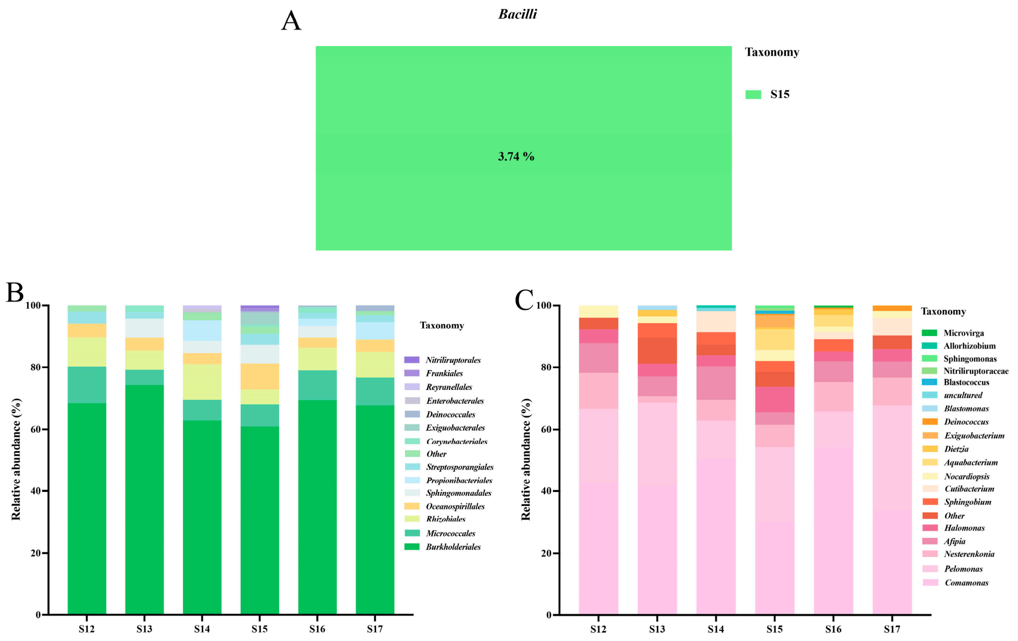

Figure S7. The relative abundance of Bacilli in control *Xoo*-infected rice (A). The relative abundances of bacterial taxa at the order level (B) and the genus level (C) of the healthy and *Xoo*-infected rice leaves treated by Cu–Ag nanoparticles and thiodiazole-copper.

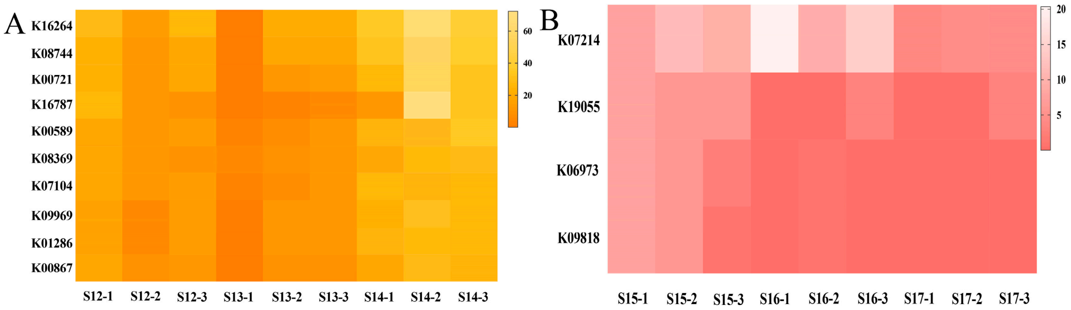

Figure S8. The heatmap demonstrates the functional prediction of genes based on the Kyoto Encyclopedia of Genes and Genomes Orthology (KO) in healthy rice (A) and infected rice (B). Each row of the heatmap represents a specific gene, while each column represents an independent rice leaf sample.

References

[1] W. Ning, X. Luo, Y. Zhang, P. Tian, Y. Xiao, S. Li, X. Yang, F. Li, D. Zhang, S. Zhang, Y. Liu, Broad-spectrum nano-bactericide utilizing antimicrobial peptides and bimetallic Cu-Ag nanoparticles anchored onto multiwalled carbon nanotubes for sustained protection against persistent bacterial pathogens in crops, Int J Biol Macromol. 265 (Pt 2) (2024) 131042. https://doi.org/10.1016/j.ijbiomac.2024.131042.

Disclaimer/Publisher's Note: The statements, opinions and data contained in all publications are solely those of the individual author(s) and contributor(s) and not of MDPI and/or the editor(s). MDPI and/or the editor(s) disclaim responsibility for any injury to people or property resulting from any ideas, methods, instructions or products referred to in the content.
